# The Beneficial Effect of *Equisetum giganteum* L. against *Candida* Biofilm Formation: New Approaches to Denture Stomatitis

**DOI:** 10.1155/2015/939625

**Published:** 2015-07-28

**Authors:** Rafaela A. S. Alavarce, Luiz L. Saldanha, Nara Ligia M. Almeida, Vinicius C. Porto, Anne L. Dokkedal, Vanessa S. Lara

**Affiliations:** ^1^Department of Stomatology (Pathology), Bauru School of Dentistry, University of São Paulo (USP), Alameda Doutor Octávio Pinheiro Brisolla 9-75, 17012-901 Bauru, SP, Brazil; ^2^Institute of Biosciences, São Paulo State University (UNESP), Botucatu, SP, Brazil; ^3^Department of Prosthodontics, Bauru School of Dentistry, University of São Paulo (USP), Alameda Doutor Octávio Pinheiro Brisolla 9-75, 17012-901 Bauru, SP, Brazil; ^4^Department of Biological Sciences, Faculty of Sciences, São Paulo State University (UNESP), Bauru, SP, Brazil

## Abstract

*Equisetum giganteum* L. (*E. giganteum*), Equisetaceae, commonly called “giant horsetail,” is an endemic plant of Central and South America and is used in traditional medicine as diuretic and hemostatic in urinary disorders and in inflammatory conditions among other applications. The chemical composition of the extract EtOH 70% of *E. giganteum* has shown a clear presence of phenolic compounds derived from caffeic and ferulic acids and flavonoid heterosides derived from quercitin and kaempferol, in addition to styrylpyrones. *E. giganteum*, mainly at the highest concentrations, showed antimicrobial activity against the relevant microorganisms tested: *Escherichia coli*, *Staphylococcus aureus*, and *Candida albicans*. It also demonstrated antiadherent activity on *C. albicans* biofilms in an experimental model that is similar to dentures. Moreover, all concentrations tested showed anti-inflammatory activity. The extract did not show cytotoxicity in contact with human cells. These properties might qualify *E. giganteum* extract to be a promising alternative for the topic treatment and prevention of oral candidiasis and denture stomatitis.

## 1. Introduction

Denture stomatitis (DS) is an inflammatory disease that affects the oral mucosa underlying a removable dental prosthesis, both partial and total. DS is considered a very common disease affecting denture wearers, especially those with maxillary complete dentures [1, 2]. Due to the strong relationship between DS and* Candida* fungus, the use of proper hygiene aids by the denture wearer is fundamental, by physical means, such as brushing, and, if necessary, chemical, and immersion of dentures in disinfectant solutions. Local or systemic antifungal therapy may still be an option in some cases of DS [3]; however, one should know the predisposing factors related to the user and eliminate them or change them whenever possible. There is no sense in using drug therapy, if it is not associated with awareness and change in the factors that contribute to DS.

Currently, treatments directed to DS include topical antifungal therapy, such as several formulations based on nystatin, systemic antifungal medication such as azoles, attention to oral hygiene, denture disinfection procedures, removal of the denture overnight, and replacement of older dentures [4].

Continued use of topical antifungal agents, nystatin suspensions [5], and disinfectants, such as sodium hypochlorite (NaOCl), glutaraldehyde, and chlorhexidine, can induce changes in the properties of the resin surface, such as roughness, hardness, and wettability, which can contribute to fungal adhesion [5–7]. The continued use of systemic antifungal therapy can also lead to serious adverse effects, such as hepatotoxicity and nephrotoxicity, and microbial resistance [4]. Moreover, after completion of antifungal therapy, there is a rapid recurrence of the DS, because the denture base resin serves as a reservoir for the fungus, and local or systemic antifungal therapies are incapable of eliminating the microorganisms present in denture bases, leading to the need of a further treatment [8].

Following this reasoning, the search for a better knowledge of antimicrobial action of medicinal plants has increased exponentially, and herbal products have proven to be an alternative to synthetic chemicals and can play an important role in the treatment of DS [9]. Studies using herbal medicines to combat dental plaque microorganisms or oral biofilm presenting* Candida* have revealed the efficacy of these agents as antimicrobial and antiadherent medications for the prevention of dental biofilm and treatment of candidiasis [10].

*Equisetum giganteum* L. (*E. giganteum*) is an endemic plant of Central and South America and is used as diuretic and hemostatic in urinary disorders and in inflammatory conditions in traditional medicine. Besides* E. giganteum* is commonly used as a substitute for* Equisetum arvense*; this drug is extensively commercialized for the purpose of beneficial role in the field of Medicine and Nutrition as a dietary supplement [11]. Although this plant has antimicrobial effect [12], until now there are no studies of* E. giganteum* against* Candida albicans (C. albicans)*.

Propolis is a resinous substance that has notable antifungal, antibacterial, antioxidant, anti-inflammatory, and immunomodulatory properties. In Odontology, the propolis has been associated mainly with the process of healing [13–17]. Considering their antifungal and anti-inflammatory activities, the propolis could be an alternative for the local treatment of DS [9, 18, 19].

Antiatherogenic [20], antitumor, antioxidant [21], and virucidal [22] effects have been reported for* Punica granatum *(*P. granatum*). The findings of studies, including some that have related the inhibition of adherence, suggest that oral bacteria and* C. albicans* are sensitive to the extract of* P. granatum* [23–25]. In a clinical study, negativity of yeasts on lesions of DS in most subjects was observed after gel application of* P. granatum*. It could be concluded that* P. granatum* extract may be used as a topical antifungal drug for the treatment of this type of candidosis [9, 26].

Therefore, in the present study, we evaluated the antimicrobial and anti-inflammatory potential of* E. giganteum* extract on* C. albicans*, as well as its influence on fungal adherence to the surface of heat-polymerized acrylic resin. The cytotoxic potential of the extract on human palatal epithelial cells and human monocytes was also assessed. This was done with a view to the future combination of the* E. giganteum* as a therapeutic/preventive alternative, for topical application, or inclusion in soft denture lining materials on inner surface of dentures, as a new alternative to the standard treatment in DS.

## 2. Materials and Methods

### 2.1. Plant Material and Extract Preparation

The aerial parts of* E. giganteum* were collected in November 2011 at the Jardim Botânico Municipal de Bauru, SP, Brazil (22°20′30′′S and 49°00′30′′W). Voucher specimens were prepared, identified, and deposited at the Herbarium of the UNESP, São Paulo State University “Júlio de Mesquita Filho”, UNBA (Bauru, SP, Brazil) under number 5795. The fresh plants were dried at 40°C for 48 h and the powdered raw material (1,3 kg) was extracted with EtOH/H_2_O (7 : 3 v/v) by percolation at room temperature. The filtrate was concentrated to dryness under reduced pressure at 40°C providing the hydroethanolic extract (70% EtOH) with a yield of 8.24% (364 g).

### 2.2. Chemical Analysis by UHPLC-PAD-ESI-MS^*n*^

The chemical profile of the 70% EtOH extract of* E. giganteum* was obtained by means of Accela High Speed LC (Thermo Scientific, San Jose, CA, USA), Thermo Scientific column (50 × 2.1 mm, 1.9 *μ*m), with PAD and coupled to an Accela (Thermo Scientific) LCQ Fleet with Ion Trap (IT) 3D and ionization by electrospray (ESI). The mobile phase consisted of ultrapure water (eluent A) and methanol (eluent B), both mixed in 0.1% of formic acid, and we utilized a proportion of 5–100 of A in B during 20 min. The experimental protocol used the following parameters: injection volume: 10.0 *μ*L; column temperature: 25°C; and flow ratio: 0.4 mL·min^−1^ and the chromatogram at 254 nm. The effluent from the UHPLC was directed into the ESI probe. The second scan event was an MS/MS experiment using a data dependent scan on deprotonated molecule [M-H]^−^ with collision energy to 10–25%. After being dissolved in MeOH-H_2_O (85 : 15), the extract was infused in the ESI source by flow injection analysis (FIA) using a syringe pump at 5 *μ*g/mL.

### 2.3. Microorganisms

For this study, we used* Staphylococcus aureus* (ATCC 6536),* Escherichia coli* (O:124), and* C. albicans* (SC 5314).* C. albicans* were grown in YEPD broth (Difco, Sparks, MD, USA) and tested in Sabouraud broth (Difco, Sparks, MD, USA). Bacteria were grown and tested in brain-heart infusion broth (BHI) (Difco, Sparks, MD, USA).

### 2.4. Antimicrobial Assay

*In vitro* antimicrobial action was analyzed by the broth microdilution method with the objective of obtaining the minimum inhibitory concentration (MIC) of the* E. giganteum* extract. Bacterial and fungal suspensions (1 × 10^5^ cells/mL) were, respectively, inoculated into BHI and Sabouraud broth using 96-well plates. Wells containing each inoculum with the 1% NaOCl solution (CTRL/NaOCl) and culture medium (Sabouraud or BHI broth) (CTRL/Medium) served as the positive and negative control, respectively.

### 2.5. Antiadherent Assay

Ninety specimens of heat-polymerized acrylic resin (2.5 × 2.0 × 0.5 cm) were fabricated according to the manufacturers' directions (Lucitone 550; Dentsply International Inc., York, USA). Each surface of specimens was abraded manually with high grade silicon carbide abrasive paper (P80; Norton Abrasivos, São Paulo, Brazil) [27], to promote a rough surface of approximately 2 microns. After this, the specimens were ultrasonically cleansed in sterile water (Ultrasonic Cleaner, Arotec, São Paulo, Brazil) [28] and sterilized with ethylene oxide (Acecil, Campinas, Brazil).

According to the different treatments, the resin specimens were divided into five groups: E50, E25, and E16 (50, 25, and 16mg/mL) of* E. giganteum *extract; CTRL/PBS, sterile phosphate buffered saline (pH 7.2); or CTRL/NaOCl-1% NaOCl solution. The treatments were performed in 6-well plates, at room temperature for 10 min. Sequentially, the specimens were incubated for 90 min at 37°C at 75 rpm (Fanem, Guarulhos, Brazil) with fungal suspension (1 × 10^7^ cells/mL), washed with PBS, and incubated in RPMI-1640 (GIBCO, Grand Island, NY) at 37°C for 12 h at 75 rpm. Three specimens for group were stained by LIVE/DEAD* Bac*Light Bacterial Viability Kit solution (Molecular Probes, Eugene, OR, USA) [29] and incubated in the dark for 15 min at room temperature.

Subsequently, the specimens were conditioned in the confocal laser scanning microscopy (Leica Microsystems GmbH, Mannheim, Germany), from which digital images were obtained. The viable cells were visualized in green, while dead cells were visualized in red. With the use of BioImage Software program, the biofilm mass was quantified including both viable and nonviable cells and expressed as *μ*m^3^. The more the biofilm mass on the specimens decreased, the higher the antiadherent activity was.

### 2.6. Cell Culture

HPEC were cultivated as previously described by Klingbeil et al. [30] and human monocytes were obtained from thirty-milliliter volumes of peripheral blood, which were drawn from apparently healthy five volunteers. After obtaining peripheral blood mononuclear cells by cell separation using Histopaque 1077 gradients, according to the manufacturer's instructions (Sigma-Aldrich Company), monocytes were counted using neutral red (0.02%) and incubated in 96-well culture plates by 2 h at 37°C in 5% CO_2_. Following, nonadherent cells were removed by aspiration. Monocytes were cultivated in RPMI-1640 (GIBCO, Grand Island, NY), supplemented with 10% FBS (GIBCO) and 1% penicillin-streptomycin (GIBCO). The medium was replaced every other day until the cells grew to about 70% confluence. The experimental protocol used in this study was approved by the Committee on Ethics in Human Experimentation, of Bauru School of Dentistry, University of São Paulo (grant number 6706412.2.0000.5417, November 8, 2012), and written informed consent was obtained from all patients. This study was performed in accordance with the Declaration of Helsinki.

#### 2.6.1. Cell Viability Assay

The different extracts were evaluated regarding cytotoxicity by using MTT (3-[4,5-dimethylthiazolyl-2]-2,5-diphenyltetrazolium bromide) colorimetric assay (Sigma Chemical Co., St. Louis, USA). Briefly, monocytes (2 × 10^5^ cells/well) suspended in 200 uL of medium were plated into 96-well tissue culture plates for 24 h. Thereafter, 200 uL of fresh medium containing various concentrations of* E. giganteum* extracts (50, 25, 16, 8, and 4 mg/mL) was added to each well and incubated for time intervals of 1, 12, and 24 hours and compared with control cells (untreated and water). After these periods, the plates were washed twice in PBS and 200 uL of fresh medium mixed with 10 uL of 5 mg/mL MTT dye in PBS was added to the wells for 4 h in a humidified 5% CO_2_ atmosphere at 37°C. After the removal of MTT solution, 150 uL of dimethyl sulphoxide (DMSO-LGCBiotecnologia, São Paulo, Brazil) was added and mixed to dissolve the dye crystals. The absorbance was measured at 500 nm by means of a multiplate reader (Synergy Mx Monochromator-Based, Biotek, Washington, DC, USA). The same assay was performed with human palatal epithelial cells (HPEC) (10^4^ cells/well) for 12 and 24 hours.

### 2.7. Measurement of Intracellular Reactive Oxygen Species (ROS)

Endogenous amounts of ROS in human monocytes were determined using the fluorescent probe 2′,7′-dichlorofluorescin diacetate (Cell Rox Deep Red Reagent; Life Technologies, Grand Island, NY, USA). Monocytes (2 × 10^5^ cells/well) were* in vitro* incubated with* E. giganteum* extract (50, 25, 16, 8, and 4 mg/mL) for 1 hour at 37°C in 5% CO_2_ atmosphere. After, they were stimulated with LPS at 5 *μ*g/mL or* C. albicans* SC5314 (1 × 10^7^ cells/mL) for three hours and incubated with 5 *μ*M of Cell Rox at 37°C in 5% CO_2_ atmosphere for 30 min. The fluorescence intensities (FIs) of the cells were measured using the multiplate reader at 640/665 nm, at 37°C.

### 2.8. Statistical Analysis

The data are expressed as the mean ± standard deviation of at least three independent experiments, in triplicate. The data were analyzed, where appropriate, by means of the one-way ANOVA, two-way ANOVA, or Kruskal-Wallis Test followed by the Tukey and Dunnett Test using the STATISTICA software program (version 12.0; StatSoft Inc., Tulsa, OK, USA). We considered *p* < 0.05 as a level of statistically significant difference.

## 3. Results

### 3.1. Compounds Isolated from* Equisetum giganteum* L

**Figure 1:**
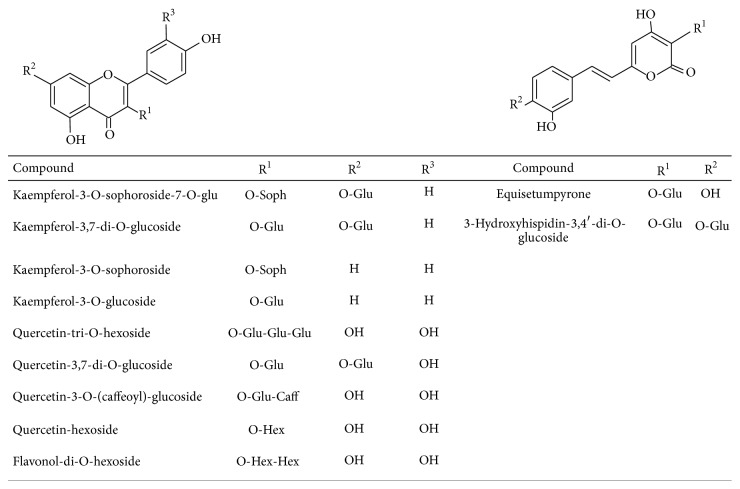
Structure of the flavonoids and styrylpyrones identified by UHPLC-PAD-ESI-MS^*n*^ in the hydroethanolic extract of* E. giganteum*.

The constituents present in the hydroethanolic extract were identified by *R*_*t*_ in RP-UHPLC, UV-vis, and MS/MS^*n*^ spectra analysis and comparison with data from the literature [11]. In total, 13 constituents were identified in the 70% EtOH extract (Table 1).  Figure 1 shows the structures of styrylpyrones and flavonoids identified.

The UHPLC-PAD-ESI-MS^*n*^ analysis of the hydroethanolic extract highlighted UV spectrum characteristic of styrylpyrones with bands at 253–258 and 355–373 nm [31] and flavonoids at 240–290 nm related to A-ring and at 300–390 nm related to B-ring [32]. Diagnostics precursor ions at* m/z* 379, 301, and 285 confirm the presence of styrylpyrones and flavonoid glucosides derivatives of quercetin and kaempferol, respectively. The signals of mass fragments at* m/z* 178 and 192 were related to presence of caffeic and ferulic acid derivatives. The neutral losses of 162 mass units allowed the identification of hexoses (sophoroside or glucoside).

**Figure 2:**
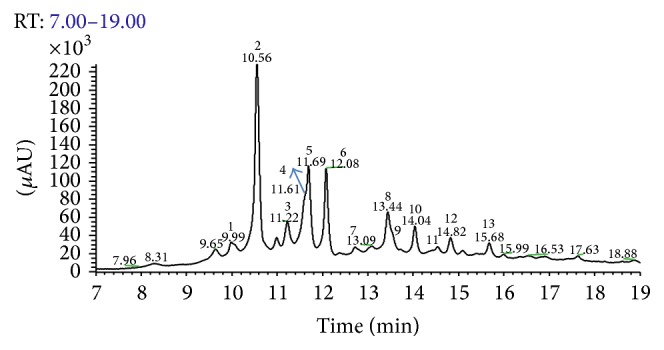
UHPLC-PAD analytical chromatogram of the 70% EtOH extract of the aerial parts of* E. giganteum* at *λ* = 254 nm. Mobile phase was water ultrapure (eluent A) and methanol (eluent B), both containing 0.1% of formic acid. The ratio was 5–100 of A in B in 20 min. Injection volume: 10.0 *μ*L; Thermo Scientific column (50 × 2.1 mm, 1.9 *μ*m); column temperature: 25°C; flow ratio: 0.4 mL·min^−1^.

In Figure 2, the UPLC-PAD chromatogram of the* E. giganteum* 70% EtOH extract is presented.

### 3.2. Antimicrobial Activity of* E. giganteum*

**Figure 3:**
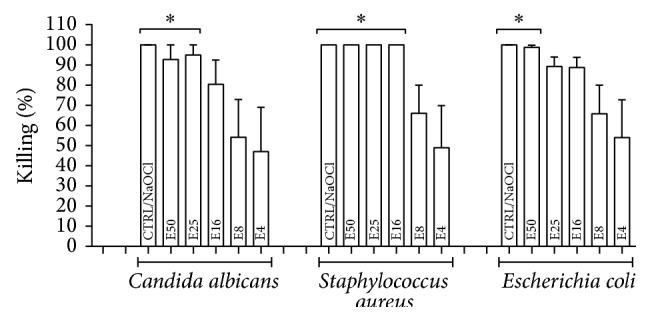
Median ± S.D. of percentage of killing against* E. coli* O:124,* S. aureus* ATCC 6538, and* C. albicans* SC 5314, after 24 hours in contact with extract at different concentrations (mg/mL): 50 (E50), 25 (E25), 16 (E16), 8 (E8), and 4 (E4). The respective controls were incubated with 1% sodium hypochlorite (CTRL/NaOCl) or with culture medium (CTRL/Medium), which showed no killing of microorganisms (0%). ^*∗*^ *p* < 0.05 represents a significant change compared with the CTRL/Medium. Five independent experiments were performed in triplicate (*n* = 15).

The* E. giganteum* extract (E50, E25, E16, E8, and E4) showed antimicrobial activity against all strains studied (Figure 3). The strain most sensitive to the* E. giganteum* extract was* S. aureus*. At the highest concentration of the extract (50 mg/mL), the results were similar to 1% NaOCl for all strains tested. Even at its lowest concentrations, the extract still showed a microbicidal action.

### 3.3. Antibiofilm Effect of* E. giganteum* against* C. albicans* SC5314

**Figure 4:**
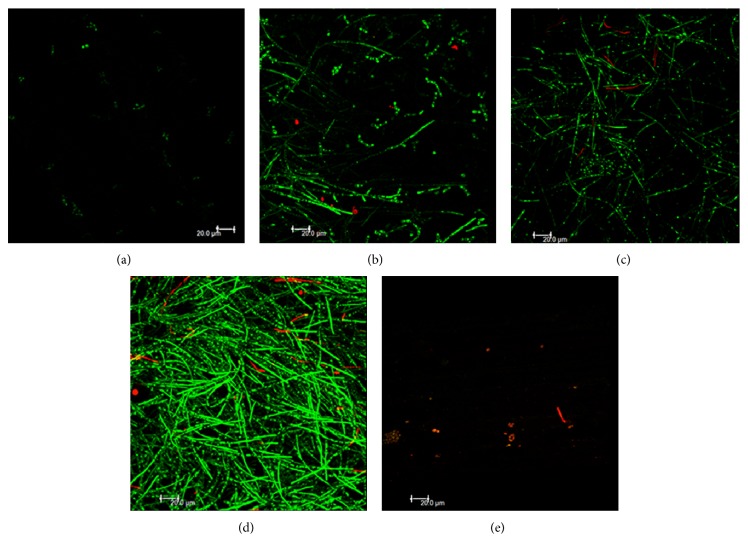
Confocal microscopy images of* Candida albicans* biofilms after each treatment with extract. (a) E50; (b) E25; (c) E16; (d) CTRL/PBS; and (e) CTRL/NaOCl. Six independent experiments were performed in triplicate.

The specimens pretreated with sterile phosphate buffered saline (CTRL/PBS) showed an expressive presence of biofilm mass of* C. albicans* on the acrylic resin specimen surface, thereby revealing biofilms at the stage of proliferation and filamentation (Figure 4(d)). As expected, this biofilm was mainly formed of viable (green) cells (Table 2). In contrast with PBS, 1% sodium hypochlorite pretreatments removed the majority of biofilm (Figure 4(e)), leaving only a few viable cells; but most of the cells were nonviable (Table 2).

The surface of resin specimens pretreated with the different concentrations of* E. giganteum* extract (50, 25, and 16 mg/mL) revealed that increasing the concentration of the extract improved its antiadherent activity, because biofilm formation decreased with an increase in amount of herbal extract used (Figures 4(a), 4(b), and 4(c)). As shown for 1% NaOCl, E50 significantly reduced the biofilm mass on the specimen surfaces, although scarce viable cells can still be noted (Figure 4(a)). All groups of the* E. giganteum* extract presented lower numbers of remaining fungal cells in comparison with control (PBS), showing marked antiadherent activity of the* E. giganteum* extracts against* C. albicans* biofilms. At 25 and 16 mg/mL, the values of biofilm mass were similar to those obtained for PBS; however, they differed statistically from both control (NaOCl) and E50. No statistical differences were observed between E50 and CTRL/NaOCl with respect to the total number of remaining nonviable cells.

### 3.4. ROS Production in Monocytes Treated with* E. giganteum* Extract

**Figure 5:**
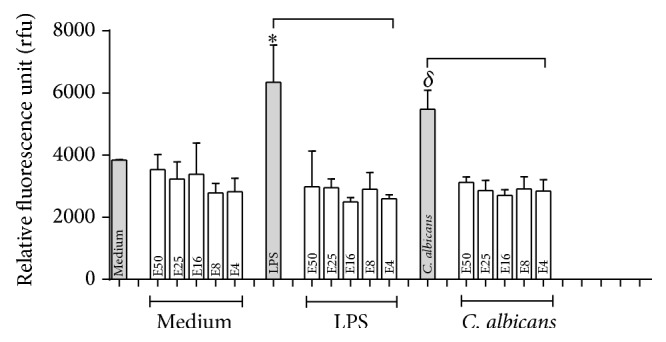
Production of ROS by human monocyte cells cultured and treated with LPS and* C. albicans* in the presence or absence of extracts as described in Materials and Methods. Results are expressed as mean ± S.D. of at least four experiments. Symbols indicate significant difference (*p* < 0.05):  ^*∗*^in comparison with LPS-treated cells and *δ* compared with* C. albicans*-treated cells, in the presence of extract in all concentrations tested.

The results revealed that all the extract concentrations (50, 25, 16, 8, and 4 mg/mL) were able to decrease the levels of ROS production by cells stimulated with LPS or* C. albicans*, returning to baseline levels. Values for cells treated with* E. giganteum* extract, which were not stimulated with LPS or* C. albicans*, remained at baseline levels (Figure 5).

### 3.5. Cytotoxicity Test

**Figure 6:**
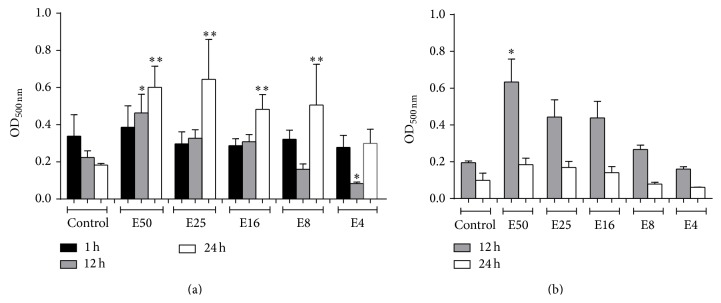
(a) Effects of* E. giganteum* on cell viability of human monocytes. The viability of cells under treatments with different concentrations of* E. giganteum* was investigated by MTT assays. Data shown are the mean ± S.D. of at least four independent experiments. Asterisks indicate significant difference compared with the control (*p* < 0.05). (b) The effects of* E. giganteum* on cell viability of HEPCs. The viability of cells under treatments with different concentrations of* E. giganteum* was investigated by MTT assays. Data shown are the mean ± S.D. of at least three independent experiments; ^*∗*^ *p* < 0.05 represents a significant change compared with the control.

Both monocytes and HPEC remained viable in the presence of* E. giganteum* extract; but, at the lowest concentration (E4) for 12 hours on monocytes, there were significant differences between the cells treated with extract and untreated cells. In addition, at highest concentrations of* E. giganteum*, the percentage of cell viability was higher than it was for untreated cells, suggesting an increased cell metabolism (Figures 6(a) and 6(b)).

## 4. Discussion

*C. albicans* is the predominant yeast species isolated from patients with DS, due to its greater capacity to form biofilms on surfaces [33–36], including denture acrylic surfaces. Therefore, decontamination of dentures is a fundamental aspect of effective oral hygiene, in order to prevent the denture stomatitis. Typically, research on* Candida* biofilms has evaluated the efficacy of different disinfectant solutions such as 1 and 2% NaOCl and 2% glutaraldehyde on heat-polymerized acrylic resin [37].

Considering that various disinfectants affect the physical properties of resin surface of denture [37–44], alternative therapies, such as phytotherapeutic agents, with antimicrobial potential and ability to eliminate or prevent the adhesion of* C. albicans* to the inner surface of the denture, without damaging the mucosa or dentures, may be of extreme interest to removable denture wearers, particularly those with maxillary complete dentures [45, 46].

In the present study, the crude extract of* E. giganteum* showed a potent antimicrobial effect on* C. albicans* and on fungus adherence to heat-polymerized acrylic resin. Furthermore, the plant extract demonstrated a potential* in vitro* anti-inflammatory effect on monocytes, while it had no cytotoxic effect* in vitro* on monocytes and HPEC. Our study is the first to verify the beneficial effect of* E. giganteum* against* Candida* biofilm formation on surfaces, and its biocompatibility.

Although* E. giganteum* does not have bacteriostatic potential, the antimicrobial action was evident in our findings, even at the lowest concentration tested (4 mg/mL). This was shown not only against* C. albicans*, but also against the* S. aureus* and* E. coli* bacteria used as parameters for comparison in our study. At a concentration of 50 mg/mL, by means of antimicrobial assay based on the broth microdilution method, the herbal extract showed antimicrobial action similar to 1% sodium hypochlorite, an agent widely applied for immersion of dentures during treatment of DS.

We emphasize the values of antimicrobial action obtained in our study, which were higher than those reported in a previous study [12] that assessed the same herbal extract against Gram-negative and Gram-positive bacteria and obtained no activity against* Escherichia coli* (*E. coli*). In our study, another aspect that highlights the significant antimicrobial activity of* E. giganteum* is that this phytotherapeutic agent was effective against a clinical strain of* E. coli* (O1124), which usually presents more resistance and higher pathogenicity than standard strains when they are isolated from patients [47]. However, even at low concentrations of the extract (8 and 4 mg/mL), we observed that its antimicrobial activity against this strain of* E. coli* ranges from 65 to 54% of death.

The chemical composition of the extract EtOH 70% of* E. giganteum* has shown a clear presence of phenolic compounds derived from caffeic and ferulic acids and flavonoid heterosides derived from quercitin and kaempferol, in addition to styrylpyrones (Table 1; Figure 1). This herbal extract produces bioactive compounds such as flavonoids that are responsible for its anti-inflammatory and antioxidant effects, in addition to having analgesic, antimicrobial, and immunomodulatory properties [48, 49]. Therefore, the flavonoids present in the composition of* E. giganteum* probably are the constituents responsible for the microbicidal effects observed here, because they have the ability to inactivate microbial adhesion proteins and transport proteins and to rupture the microbial membrane.

Furthermore, in order to simulate the resinous portion of removable dentures that have been contaminated by* C. albicans*, as frequently it occurs with dentures under the conditions of the intraoral environment, we developed contaminated specimens made of denture base material. The resin surface pretreated with extract of* E. giganteum* at all concentrations showed a significant reduction in the biofilm mass of viable* C. albicans* cells in comparison with the untreated resin. In other words, we can assert that a brief 10-minute treatment of the resin surface with the extracts was able to significantly reduce the capacity of* C. albicans* to adhere to the surface. We emphasize that, at 50 mg/mL, the treatment with* E. giganteum* resulted in the lowest biofilm mass found, when compared with any of the other concentrations. Interestingly, after these pretreatments, the resin surface showed dispersed fungus in both its filamentous and yeast forms, especially at the highest concentration of the extract. As observed in our control samples, confluent biofilms presenting predominantly filamentous forms are developed on the resin surface. Taken together, we believe that the herbal extract was able to interfere in the expression of fungal virulence factors, in addition to having an antiadherent effect on the microorganism.

One possible hypothesis for explaining the effect of biofilm removal by* E. giganteum* extract could be based on biofilm pH. Since the most of biofilms are formed at neutral pH, the conditions having very high or very low pH values can provide changes on microbial metabolism and surface properties, interfering in their adhesion process to surfaces [50]. Hypochlorite solutions, for example, having alkaline pH (pH > 11), can increase the electrostatic repulsion between the yeast cells and the surface of the resin material, dissolving the biofilm cells [51]. Thus, the acidic pH (pH = 5.48) of the hydroalcoholic extract of* E. giganteum*, associated with antimicrobial activity, especially in higher concentrations, could explain their important antiadherent effect on* C. albicans* biofilms; however, further studies are required about the mechanisms involved.

In addition to the beneficial results with respect to the antimicrobial and antiadherent activities, all concentrations of* E. giganteum* extract demonstrated suppression of reactive oxygen species in human monocytes stimulated* in vitro* by LPS or* C. albicans*. Previous studies have also shown reduced levels of ROS resulting from the action of* E. arvense* and have attributed this effect to the presence of flavonoids [52]. In fact, flavonoids are able to eliminate ROS and RNS by donation of electron and hydrogen (H^+^) from the hydroxyl groups present in their composition, stabilizing these free radicals [49, 53] or suppressing the formation of ROS by inhibiting the enzymes involved in generating them [49, 54]. The presence of phenolic acids was also detected in the* E. giganteum* extract. This group of substances is characterized by their antioxidant properties, since they were able to reduce the levels of reactive oxygen species due to trapping free radicals directly or scavenging them through a series of combined reactions with antioxidant enzymes [49, 52, 53, 55]. Other inflammatory mediators should be evaluated for a more accurate conclusion and to imply that* E. giganteum* extract could negatively modulate monocyte-mediated inflammatory responses.

Another interesting finding was that the extract did not alter the viability of monocytes and HPEC for up to 24 hours. This has important clinical repercussions, since these cells are closely related to the inner surface of the denture, in denture wearers. The oral epithelial cells are the first cells to interact with* C. albicans* during the establishment of DS.

In summary, our results suggest that the phytotherapeutic* E. giganteum* extract has the following properties: antimicrobial against* C. albicans*,* S. aureus,* and* E. coli*; antiadherence of* C. albicans* to heat-cured acrylic resin specimens; and anti-inflammatory effects on human monocytes activated by* C. albicans*. In addition, the phytotherapeutic extract did not compromise the viability of human monocytes or human palatal epithelial cells. These properties might qualify* E. giganteum* extract to be a promising alternative for the topic treatment and prevention of oral candidiasis and denture stomatitis.

## Figures and Tables

**Table 1:** UHPLC-PAD-ESI-MS data (UV-vis spectra and detected ions) and MS^*n*^ (product ions) of compounds detected in EtOH 70% of the aerial parts of *E. giganteum*.

Peak	*R*_*t*_ (min)	UV (*λ*_max_)	UHPLC-MS ions [M-H]^−^	ESI-IT-MS^*n*^	Compound
1	9.99	266, 350	787	463, 301	Quercetin-tri-*O*-hexoside
2	10.56	265, 344	771	609, 428, 285	Kaempferol-3-*O*-sophoroside-7-*O*-glu
3	11.22	254, 366	585	422, 379, 259	3-Hydroxyhispidin-3,4′-di-*O*-glucoside
4	11.61	218, 295sh, 327	625,07	463, 301	Quercetin-3,7-di-*O*-glucoside
5	11.69	278, 305sh, 327	625,01	463, 301, 255, 178	Quercetin-3-*O*-(caffeoyl)-glucoside
6	12.08	264, 341	609	447, 327, 285, 255	Kaempferol-3,7-di-*O*-glucoside
7	13.09	270, 361	625	462, 301	Flavonol-di-*O*-hexoside
8	13.44	254, 331, 369	423	379, 287, 261, 217	Equisetumpyrone
9	13.53	216, 291, 328	—	215, 178, 160, 142	Caffeic acid derivative
10	14.04	265, 287sh, 341	609	285	Kaempferol-3-*O*-sophoroside
11	14.55	270, 347	463	301	Quercetin-hexoside
12	14.82	275, 297sh, 331	463	309, 192, 177, 133	Ferulic acid derivative
13	115.68	265, 344	447	285	Kaempferol-3-*O*-glucoside

**Table 2:** Mean ± S.D. of biofilm cells (biofilm mass), including those that are viable and nonviable (mean), remaining on the surface of the specimens after different pretreatments with extract: 50 mg (E50), 25 mg (E25), or 16 mg (E16), and PBS (CTRL/PBS) or 1% sodium hypochlorite (CTRL/NaOCl), for 10 minutes. The data were obtained by using the confocal laser scanning microscopy and BioImage software program. Six independent experiments were performed in triplicate (*n* = 18).

Groups	Biofilm mass (*µ*m^3^)	(Viable + nonviable)
E50	4.263,03 ± 4.037,27	(4.231,74 + 31,29)
E25	31.408,34 ± 5.552,14	(28.825,29 + 2.583,05)
E16	35.448,57 ± 4.950,06	(34.063.22 + 1.385,35)
CTRL/PBS	95.488,84 ± 40.389,17	(94.060,39 + 1.428,45)
CTRL/NaOCl	489,36 ± 244,11	(386,17 + 103,19)
